# Prevalence of Rubella Antibodies among Fertile Women in the West of Romania, 18 Years after the Implementation of Immunization

**DOI:** 10.3390/vaccines9020104

**Published:** 2021-01-29

**Authors:** Florin Gorun, Daniel Malita, Ioana Ciohat, Tatjana Vilibic-Cavlek, Horea Feier, Irena Tabain, Marius Craina, Octavian Cretu, Dan Navolan

**Affiliations:** 1 Department of Obstetrics-Gynecology, “Victor Babes” University of Medicine and Pharmacy Timisoara, Eftimie Murgu Square nr. 2, 300041 Timisoara, Romania; gorun.florin@umft.ro (F.G.); craina.marius@umft.ro (M.C.); navolan@umft.ro (D.N.); 2 Department of Radiology, “Victor Babes” University of Medicine and Pharmacy Timisoara, Eftimie Murgu Square nr. 2, 300041 Timisoara, Romania; malita.daniel@umft.ro; 3 Laboratory of Antenatal Medicine, City Unversitary Emergency Hospital Timisoara, str. Odobescu, nr. 1-3, 300202 Timisoara, Romania; ioana.ciohat@umft.ro; 4 Department of Virology, Croatian Institute of Public Health, Rockefeller str. 12, 10000 Zagreb, Croatia; irena.tabain@hzjz.hr; 5 School of Medicine, University of Zagreb, Salata 3, 10000 Zagreb, Croatia; 6 Department of Cardiovascular Surgery, “Victor Babes” University of Medicine and Pharmacy Timisoara, Eftimie Murgu Square nr. 2, 300041 Timisoara, Romania; horea.feier@umft.ro; 7 Department of Surgery, “Victor Babes” University of Medicine and Pharmacy Timisoara, Eftimie Murgu Square nr. 2, 300041 Timisoara, Romania; octavian.cretu@umft.ro

**Keywords:** rubella virus, epidemiology, vaccine, pregnant women, Romania

## Abstract

Seronegative women are susceptible to primary rubella virus (RV) infection during pregnancy, which can cause fetal damage. Vaccination represents the main strategy in rubella prevention. The aim of this study was to analyze changes in the rubella seroprevalence and identify populations with a high susceptibility to RV. A cross-sectional study was performed on 6914 Caucasian fertile women who had *Toxoplasma gondii*, other viruses, Rubella, Cytomegalovirus, and the herpes simplex virus (TORCH) screening in two distinct periods—1452 at the Timișoara Municipal Hospital, Romania (Group 1: 2008–2010) and 5462 at the laboratory Bioclinica S.A., Timișoara, Romania (Group 2: 2015–2018). The RV seroprevalence decreased (Group 1 versus Group 2; 94.1% (92.7–95.2) versus 91.4% (90.6–92.1), OR = 0.76 (*p* = 0.0007)). According to the year of birth and eligibility to vaccination program, RV seroprevalence rates were 82.4% (76.8–86.8)/1997–2004, 85.4% (80.5–89.3)/1995–1996, 90.1% (89.0–91.1)/<1989, and 95.8% (94.7–96.6)/1989–1994. No significant difference in the RV seropositivity according to the place of residence was found. The overall RV susceptibility increased from 2008–2010 to 2015–2018. The highest susceptibility was found in women born between 1997–2004 eligible for measles-mumps-rubella (MMR) vaccine through the family practice system and the lowest in women born between 1989–1994 eligible for monovalent rubella vaccine conducted in schools.

## 1. Introduction

Rubella, also known as German measles, is a contagious disease caused by the rubella virus, a positive-stranded RNA virus, that belongs to the *Togaviridae* family and the only member of the *Rubivirus* genus [1]. Viral particles measure 50–85 nm in diameter. The viral genome encodes three structural proteins (capsid protein, glycoproteins E1 and E2). Humoral antibody responses are directed against the E1 glycoprotein [1,2]. Humans are the only known reservoir of infection [2,3]. Rubella virus is transmitted from person to person through the aerosol route, immunity after infection being present throughout life. In non-pregnant women, the rubella virus usually leads to a mild, self-limiting infection associated with a characteristic rash, while 25–50% of infected patients are asymptomatic [3,4]. However, infection during pregnancy, especially in the first trimester, can cause miscarriage or birth of an infant with congenital rubella syndrome (CRS) [1,5]. In an infected pregnant woman, following placental infection, the virus can spread through the developing vascular system of the fetus [3], causing infection. Not all women infected during pregnancy transmit the infection vertically. The risk of transplacental transmission correlates with the gestational age at which pregnant women acquire the infection—up to 90% in the first 12 weeks of pregnancy, 54% in weeks 13–14, and 25% by the end of the second trimester of pregnancy [5]. Clinical manifestations of CRS include hearing impairments (sensorineural hearing loss), eye anomalies (cataracts, retinopathy, microphthalmia, pigmentary and congenital glaucoma), heart defects (patent ductus arteriosus, pulmonary stenosis, ventricular septal defect), and other lifelong disabilities (developmental delay, autism, encephalitis, diabetes mellitus, thyroiditis) [5,6]. Approximately 25% of children with CRS syndrome have congenital cataracts, and in 50% of cases this is bilateral (3). Approximately 20% of the patients have persistent arterial canal and 12% have peripheral pulmonary artery stenosis. Hearing impairment may be the sole manifestation of CRS, being present in approximately 60–75% of cases [3,4]. Complications of CRS in the neonatal period include prematurity, intrauterine growth retardation, purpuric rash, microcephaly, hemolytic anemia, hepatomegaly, splenomegaly, “blueberry muffin” spots, hypotonia, bulging anterior fontanelle, constricted maxillary arch, high palate, interstitial pneumonia, myocarditis, myositis, nephritis, and meningoencephalitis [3]. A report from 2015 showed that around 100,000 children are born with CRS worldwide each year (1). The highest risk of CRS is in countries where fertile women do not have immunity against rubella [6]. The highest rate of seronegativity is recorded in the East Asian region (19.4%), followed by the African region (10.7%) and the American region (9.7%) [7]. There is no specific treatment for rubella and CRS, but they can be prevented by vaccination. By December 2018, 168 of 194 countries had introduced rubella vaccines and global coverage was estimated at 69%. The lowest coverage was found in Africa and Southeast Asia, where the CRS rate is highest [6]. However, rubella reinfection is possible after natural infection or successful immunization due to being typically subclinical. In reinfection during pregnancy, the risk of transmission to the fetus is difficult to determine but some studies have shown that the risk of CRS is most likely below 5% [2].

Most licensed rubella vaccines contain a live attenuated virus strain, and a single dose gives over 95% immunity, which is similar to that induced by natural infection [8]. After two doses of MMR, the seroconversion rate is approximately 99% and the rubella-specific antibodies may even persist through lifetime [1]. The most commonly used rubella vaccines are based on live attenuated strains—RA 27/3, TO-336, and BRD-2 [5]. Rubella vaccines are available either as a monovalent formula or in combination with other vaccines. The most commonly used rubella-containing vaccines (RCVs) are combinations with measles (MR), measles and mumps (MMR), or measles, mumps, and varicella (MMRV) vaccines [6,8]. At least five MMR vaccines are available, which include (1) Triviraten (Berna Biotech Pharma GmbH, Bern, Switzerland) (EZ 19 measles strain; Rubini mumps strain; Wistar RA 27/3 rubella strain propagated on human diploid cells), (2) M-M-R Merck (Merck & Co. Inc., Whitehouse Station, NJ, USA) (Enders attenuated Edmonston measles strain propagated in chick embryo cell culture; Jeryl Lynn mumps strain propagated in chick embryo cell culture; Wistar RA 27/3 rubella strain propagated on human diploid lung fibroblasts), (3) Morupar Chiron (Chiron Corporation, Emeryville, CA, USA) (Schwarz measles strain propagated in chick embryo cell culture; Urabe AM 9 mumps propagated in chick embryo cell culture; Wistar RA 27/3 rubella strain propagated on human diploid lung fibroblasts), (4) Priorix vaccine (GSK, London, England, UK) (attenuated Schwarz measles; RIT 4385 mumps strain; Wistar RA 27/3 rubella strain), and (5) Trimovax (Sanofi Pasteur, Lyon, France) (Schwarz measles strain; Urabe AM 9 mumps strain; Wistar RA 27/3 rubella strain) [9]. MMR vaccine is a part of CDC Recommended vaccinations for infants and children administered at 12–15 months and 4–6 years old [10].

**Figure 1:**
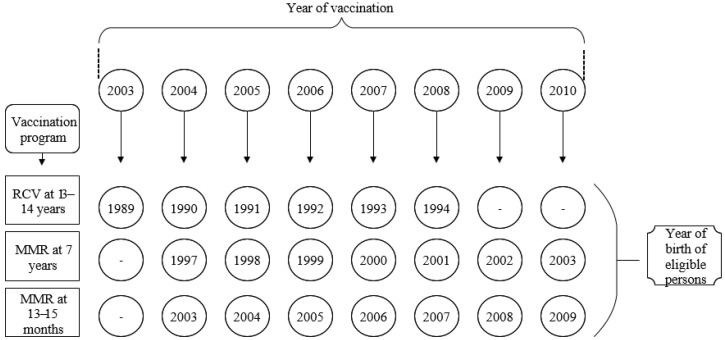
Romanian rubella national vaccination schedule (2003–2010). RCV = Monovalent Rubella contained vaccine; MMR = Measles-Mumps-Rubella vaccine.

Romanian national vaccination program included monovalent RCV from 2003 to 2008 and combined MMR vaccine starting from 2004 (Figure 1). At present, MMR is administered to 12–15 months old and five-year-old children. The national vaccination program did not include anti-rubella vaccination for women born in 1988 and before, respectively, and those born in 1995–1996. For these women, anti-rubella vaccination was opportunistic.

The aim of this study is to analyze the evolution of rubella seroprevalence among fertile women tested in two intervals (2008–2010 and 2015–2018). We also aimed to identify populations with high susceptibility to rubella, according to the place of residence, age, year of birth, and eligibility for the national immunization program.

## 2. Materials and Methods

### 2.1. Participants and Study Design

A cross-sectional study was performed on 6914 Caucasian fertile women (14–45 years old) who had *Toxoplasma gondii*, other viruses, rubella, cytomegalovirus, and the herpes simplex virus (TORCH) screening in two distinct periods—1452 at the Timișoara Municipal Hospital, Romania (Group 1: 2008–2010) and 5462 at the private laboratory BIOCLINICA S.A., Timișoara, Romania (Group 2: 2015–2018). The women residing in one of the five counties in the western region of Romania (Timis County, Caras–Severin County, Arad County, Hunedoara County, and Bihor County). Only women who declared their place of residence (urban or rural) were included.

### 2.2. Variables and Methods

IgG-and IgM-anti-rubella antibodies were determined in serum samples of all participants. Features such as the presence of rubella antibodies, age, year of birth, and place of residence of the participants were included in the analysis. No personal history of vaccinations was reported.

The titer of IgG-anti-rubella antibodies and IgM-anti-rubella antibodies was determined by the chemiluminescence method (CLIA) by an Immulite One Machine (Diagnostic Products Corporation, Los Angeles, CA, USA) and commercial tests (Siemens Healthcare Diagnostics Products, Llanberis, Gwynedd, United Kingdom) for the first group of patients (2008–2010) and by chemiluminescent microparticle immunoassay (CMIA) method using an Architect i1000SR engine (Abbott Park, Illinois, Chicago, IL, USA) and commercial tests (Abbott, Max-Planck-Ring 2, Wiesbaden, Germany) for the second group of patients (2015–2018).

The distribution of anti-rubella IgG titers was evaluated as one of three categories— negative titer (<5 IU/mL), inconclusive titer (5–10 IU/mL), and positive titer (>10 IU/mL). In the assessment of the rubella immunity, inconclusive serology results were considered negative. Participants with IgM-anti-rubella-positive results were excluded. The demographic data of the participants, such as year of birth, age, and place of residence were collected from the completed forms at the time of testing.

### 2.3. Data Analysis Procedure

The data were stored in the Astraia database (Astraia Software GmbH, Munchen Germany) and Microsoft Office Excel (Microsoft Corporation, Redmond, WA, USA) software.

The following parameters were analyzed: the overall seroprevalence of rubella and its evolution between the two intervals (2008–2010 and 2015–2018) and the association of seroprevalence with the place of residence, respectively, the age of women, and their year of birth. The place of residence was defined as urban or rural according to the administrative division of Romania.

For the analysis of seroprevalence according to the year of birth, the participants were divided into several cohorts depending on the eligibility to the national vaccination program. The participants included in the first group had the year of birth between 1966 and 1994 and were divided into two cohorts—born before 1989 (ineligible for the national vaccination program) and born between 1989 and 1994 (eligible for the monovalent RCV national vaccination program). Patients included in the second group had the year of birth between 1970 and 2004 and were divided into four cohorts—born before 1989 (ineligible for the national vaccination program), 1989–1994 (eligible for the monovalent RCV national vaccination program), 1995–1996 (ineligible), and after 1997 (eligible for MMR vaccines).

GraphPad Prism 8.0.2 (GraphPad Software, Inc. 2365 Northside Dr. Suite 560, San Diego, CA, USA) software was used for statistical evaluation. Ficher’s exact test was used to compare the proportions. *p* < 0.05 was considered significant. Baptista–Pike method was used to compute confidence intervals for the odds ratio. The Wilson–Brown method was used to calculate the confidence intervals for the seroprevalence.

## 3. Results

### 3.1. Demographic Characteristics of Study Participants

Of the 1452 participants included in Group 1 (2008–2010), 72.0% (*n* = 1046) come from urban areas and 28.0% (*n* = 406) from rural areas. Of the 5462 women included in Group 2 (2015–2018), 64.9% (*n* = 3543) come from urban areas and 35.1% (*n* = 1919) from rural areas.

The age of the participants in the first group (2008–2010) varied between 14 years old and 43 years old, median 28 years old (interquartile interval = 6), and their year of birth was between 1966 and 1994. The participants in the second group (2015–2018) were aged between 14 years old and 45 years old, median 29 years old (interquartile interval = 7), and their year of birth was between 1970 and 2004.

### 3.2. Seroprevalence of Rubella among Fertile Women

**Figure 2:**
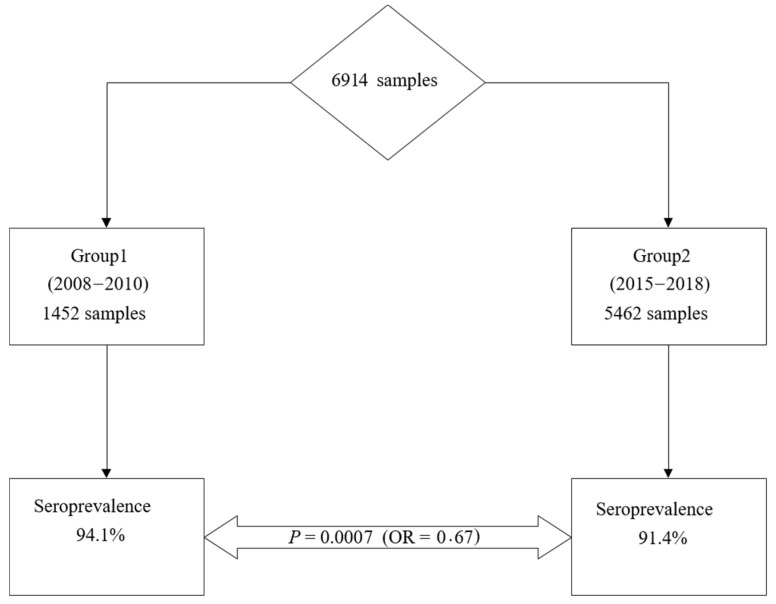
Evolution of rubella seroprevalence among fertile women.

Seroprevalence of rubella was 94.1% (*n* = 1366/1452; 95% CI = 92.7–95.2) among women included in the first group and 91.4% (*n* = 4994/5462; 95% CI = 90.6–92.1) in women in the second group, respectively. The rubella seroprevalence among fertile women decreased significantly from 2008–2010 to 2015–2018, *p* = 0.0007; OR = 0.67; 95% CI = 0.47–0.85 (Figure 2).

In the first group, 5.4% (*n* = 78/1452; 95% CI = 4.3–6.7) of women were seronegative and 0.5% (*n* = 8/1452; 95% CI = 0.3–1.1) had an inconclusive titer, while in the second group, 6.9% (*n* = 376/5462; 95% CI = 6.2–7.6) of women were seronegative and 1.7% (*n* = 92/5462; 95% CI = 1.4–2.1) had an inconclusive titer.

### 3.3. Seroprevalence of Rubella among Fertile Women According to Demographic Parameters

Results for women included in the first group (2008–2010) indicated a seroprevalence of 94.2% (*n* = 985/1046; 95% CI = 92.6–95.4) in urban areas and 93.8% (*n* = 381/406; 95% CI = 91.1–95.8) in rural areas. In the participants from Group 2 (2015–2018), the seroprevalence was 91.6% (*n* = 3247/3543; 95% CI = 90.7–92.5) in urban areas and 91.0% (*n* = 1747/1919; 95% CI = 89.7–92.2) in rural areas. There was no statistically significant difference between the two areas of residence (urban/rural) in both studied groups—*p* = 0.80, OR = 0.94 (95% CI = 0.58–1.50) in Group 1 and *p*= 0.44, OR = 0.92 (95% CI = 0.50–1.12) in Group 2.

As regards the stratification by year of birth, we recall that the participants included in the two groups were divided into several cohorts according to the eligibility for the national immunization program.

The participants included in Group 1 were divided into two cohorts—born before 1989 and between 1989 and 1994. After stratification in cohorts, no statistically significant difference between the seroprevalence of women in urban and rural areas was found. Women born between 1988–1994 eligible for the RCV vaccine through the program conducted in schools had higher seroprevalence values than women born before 1989 (no-eligible in the national immunization program) (Table 1).

The participants included in Group 2 were divided into four cohorts—born before 1989, 1989–1994, 1995–1996, and after 1997. No statistically significant difference between the seroprevalence of women in urban and rural areas was found, either as a whole or in individual cohorts.

When analyzed independently from the place of residence, the highest rubella seroprevalence was found in women born between 1989–1994 who were eligible for monovalent RCV vaccine through the program conducted in schools, followed by women born before 1989, women born in 1995–1996 (non-eligible for vaccination), and women born between 1997 and 2004, who were eligible for MMR vaccination conducted through a family practice system. A similar trend was found in women from rural areas. Interestingly, in urban areas, both cohorts of women non-eligible for national vaccination program born before 1989 and in 1995–1996 had similar seroprevalence rates.

The rubella seroprevalence, depending on the age of the participants was calculated (Table 2). Seroprevalence was stable in each age subgroup. No correlation between seroprevalence and age was found in both groups (2008–2010 and 2015–2018).

## 4. Discussion

This is the largest study on the seroprevalence of TORCH infections among women of reproductive age in Romania [11,12]. The presented results show that the rubella seronegative rate among women of reproductive age in the western region of Romania increased between 2008–2010 and 2015–2018 from 5.9% to 8.6%, above the European average of 7.6% [7]. Several causes could explain the increase of seronegative rates, such as a decrease in vaccination coverage or the gradual disappearance of the virus from the community due to improved hygiene. In European countries, this rate varies between 1.4% in the Czech Republic and 45% in Turkey [7]. In epidemiological surveys conducted among European childbearing women, the seroprevalence of rubella has been reported to be 97.9% in Germany, 99.8% in Poland, 80.6% in Italy, and 94.6% in Croatia [13,14,15]. Seroprevalence of rubella similar to that of reproductive-aged women included in Group 2 (tested between 2015–2018) of our study (91.43%) was reported in Cyprus (90.3%), Hungary (91.8%), Latvia (91.3%), and Luxembourg (91.9 %) [16].

World Health Organization (WHO) has set the target for eradication of measles and rubella in at least five WHO regions by 2020 [17]. One of the regional strategies adopted by WHO to eradicate measles and rubella is to obtain and support very high coverage (≥95%) with two doses of measles vaccine and at least one dose of rubella vaccine through routine immunization services. All countries in the European Union have introduced two doses of MMR vaccination in their national vaccination schedule. The number of WHO state members that included the rubella-containing vaccine in their immunization schedules in 1996 was 83 of 193 (44%). The number increased to 141 out of 194 (72.7%) in 2014 and 152 out of 194 (78.4%) in 2016 [2]. In 2017, vaccination coverage for the first dose of rubella-containing vaccine was between 85% and 95% in countries such as Bulgaria, France, Italy, UK, Croatia, Poland, Lithuania, The Netherlands, Iceland, Estonia, Cyprus, and Finland, and over 95% in Spain, Portugal, Germany, Hungary, Greece, Austria, Slovakia, Czech Republic, Belgium, Sweden, Norway, Latvia, and Luxembourg [18]. Romania was the only country in the EU/EAA member states where the coverage (in the year 2017) with the first dose of rubella vaccine was below 85% [17]. In Romania, the MMR vaccine coverage decreased from 93.2% in 2010 to 85.8% (dose I) and 67% (dose II) in 2015. In 2018, the MMR vaccine coverage was 89.6% and 80.9% for dose I and dose II, respectively.

The number of rubella cases in the European Region declined from 29,617 in 2012 to 2437 in 2015. It should be noted that out of the 28,536 cases reported in 2012 in the 26 countries of the European Union and the European Economic Area, 99% were from Poland and Romania [2]. In 2015, the elimination target was reached in 14 countries of the European Union (EU) [19]. In 2017, 37 countries (70%) from the WHO European Region provided evidence to demonstrate the elimination of endemic rubella (interrupted transmission for at least 36 months). Interrupted endemic transmission for 24 months was reached in two European countries (4%) and interrupted endemic transmission for 12 months was reached in three European countries (5%). However, in eight European countries (8%), there is endemic transmission [20]. Between March 2019 and February 2020, 376 cases of rubella were reported, of which 38 (10%) were confirmed by the laboratory in nine EU Member States and the United Kingdom. Eighteen countries did not report cases during this 12-month period [21]. In Romania being confirmed four cases during this period [21].

In Romania, in 2010, the incidence of rubella in women of reproductive age was 0.5/100,000 (age group 15–19 years old), 0.1/100,000 (age group 20–24 years old), 0.4/100,000 (age group 25–34 years olf), and 0.1/100,000 (age group 35–44 years old) [22]. The incidence has decreased, no case of rubella infection was registered in pregnant women in Romania in 2018, with the exception of one case reported in women of reproductive age (37 years old) [23]. Regarding CRS, during 2010, a number of 47 possible cases of CRS were reported [22]. In 2018, 11 suspicious cases of CRS were reported—10 probable cases and one denied case. The incidence of probable cases of CRS in 2018 was 5.8 per 100,000 live births, and no deaths were recorded [23]. The number of confirmed rubella cases decreased in Romania from 145 in 2013 to 9 in 2017 [24].

The last rubella epidemic in Romania occurred between September 2011 and December 2012. A total of 24,627 cases were registered at the national level, with an incidence of 97.3/100,000 inhabitants [25]. The monthly incidence of rubella cases started to increase in September 2011, reaching a maximum value (36.04/100,000) in March 2012, after which it decreased to 0.03/100,000 in December 2012. One of the highest incidence rates in 2011 was found in Bihor county (31/100,000 inhabitants), located in western Romania. The highest incidence during the epidemic was reported in February 2012 (43.97/100,000 inhabitants) [25]. In March 2012, the incidence in the age group 15–19 years old was 386.4/100,000 inhabitants. During the epidemic, of the total cases of rubella in females (*n* = 10,134), a percentage of 93.7% (*n* = 9501) was recorded among 14-year-old girls and women of childbearing age (age group 15–49 years old) [25]. There were 119 cases among pregnant women. The highest infection rate occurred among people born in 1995–1996 (*n* = 8694, 35.3%) who were not eligible for the vaccination program. In our study, women born during this period have a seronegative rate of 14.6%. In 2012, the incidence of confirmed cases of CRS was 11.93/100,000 live births, and 11 deaths and one stillbirth were recorded.

In our study, the highest seroprevalence was found among women born in 1989–1994 (95.8%), eligible for RCV vaccination at 14–15 years old. Moreover, the lowest seroprevalence is found in women born after 1997, eligible for MMR at 7 years old or 13 months old. According to the Romanian national vaccination program, the MMR vaccine has been introduced for seven-year-old girls born since 1997 and for 13- to 15-month-old girls born since 2003. This vaccination has been performed through the family practice system compared to the RCV program that was conducted in schools. Since the introduction of the MMR combined vaccine in the national calendar, vaccine coverage has been maintained for more than 95% (WHO target). Governmental data showed that the vaccination coverage decreased from 93.2% in 2010 and stagnated at 89.6% (dose 1) and 80.9% (dose 2) in 2018. Taken together, the decrease in the seroprevalence, respectively, in vaccination coverage in the same period (2010–2018) argues for the necessity of improving the vaccination uptake in the population.

The spread of vaccine refusal has become a risk factor for rubella outbreaks. A possible cause of the parents’ refusal to vaccinate their children could be due to lower confidence in the benefits of vaccination. Strong predictors of low vaccination coverage may also be misleading knowledge, beliefs, and perceptions about vaccines, generally negative attitudes and behaviors toward vaccination, and low socioeconomic status such as especially low incomes and education, a large number of children, and unmarried status [26]. The most damaging controversy over vaccine safety concerns a speculative hypothesis that the MMR vaccine may be a cause of autism. Wakefield et al. were the first to expose the possibility of the MMR vaccine causing autism in 1998 [27]. This controversy started with the exploration of the possible role of measles and measles vaccines in the pathogenesis of inflammatory bowel disease. Following the publication of Wakefield’s paper in 1998, numerous scientific studies have rejected the link between the MMR vaccine and autism. However, some parents are still reluctant to accept MMR vaccination of their children because they are unsure of the safety of the vaccine [27]. Wakefield’s article linking MMR vaccine and autism was retracted for fraud, but this misinformation persists [28,29]. The falsified report continues to cause parental concern; therefore, physicians should inform parents about the benefits of immunization, the risks of infection, the safety of the vaccine, complications, and possible risks. In addition, physicians must inform parents that a vaccine is not without risks and side effects. Available MMR vaccines are usually well-tolerated, side effects are often benign and include fever, lymphadenopathy, rash, or parotitis. Febrile seizures, anaphylaxis, thrombocytopenic purpura, or encephalitis are rarely reported adverse reactions [2]. MMR vaccines containing Urabe and Leningrad-Zagreb mumps strains have been associated with aseptic meningitis [9].

WHO created the “SAGE Working Group on Vaccine Hesitancy” to characterize, discuss, and establish strategies to address issues related to vaccine refusal. The first tasks set in 2012 were to propose a definition of hesitation and its scope and to develop a model to classify the factors influencing the behavioral decision to accept a vaccine [30]. Vaccination hesitancy refers to delay in acceptance or refusal of vaccines despite the availability of vaccine services. Vaccine hesitancy has been reported in more than 90% of countries in the world. For example, in the UK, coverage of the MMR vaccine decreased to 91.2%, the lowest level since 2011–2012, and in the USA, the percentage of children aged 19–35 months who received the MMR decreased from 91.6% in 2011 to 91.5% in 2017. Media platforms (including social networks) have had a huge influence on the spread of vaccine hesitation, the main problem being misinformation, according to Anthony Fauci, director of the National Institute for Allergies and Infectious Diseases, USA [31]. The WHO SAGE Working Group arranged determinants of vaccine hesitancy in three categories—contextual (influences arising due to historic, socio-cultural, environmental, health system/institutional, economic, or political factors), individual (influences arising from personal perceptions of the vaccine or influences of the social/peer environment and group) and vaccine/vaccination-specific influences (directly related to vaccine or vaccination) [32]. The major goal of rubella vaccination is the prevention of congenital infection, the time required to eliminate rubella and CRS completely vary from less than 10 years to 30 years, depending on the strategy used [2].

Public health campaigns in the WHO Region of the Americas have shown that the transmission of the rubella virus can be interrupted. In the USA, the current live strain of rubella vaccine authorized for use is the RA27/3 strain, first isolated in the 1960s [1]. Two doses, the first at 12–15 months of age and the second at 4–6 years of age are included on the USA childhood immunization schedule [33]. The rubella vaccine was introduced in 1969 before licensure rubella was common [2,34]. In the pre-vaccination era, epidemics occurred every six to nine years and incidence of CRS varied from 0.1–0.2 per 1000 live births during endemic periods and from 0.8–4.0 per 1000 live births during epidemics periods [1,34]. After the introduction of the rubella vaccine, the number of rubella cases in the United States decreased from 57,686 to 12,491 between 1969 and 1976 [34]. The Pan American Health Organization announced the elimination of rubella from the Americas in 2015 [35].

No correlation between age and rubella seroprevalence among fertile women was found in our study, similar to Croatia, Bulgaria, and Slovenia [15,16]. Some countries such as Belgium, England, and Wales showed an increase in seroprevalence of rubella with age, while Hungary and Lithuania reported a decrease in seroprevalence with age [16].

The main strength of our study consists of the sample size. This is the largest study on rubella epidemiology in Romania. Stratification of participants according to several parameters—year of birth, age, and place of residence—allows for more precise identification of high-risk population.

Our study has several limitations, however. It was not designed to assess participants’ vaccination status, so no data were collected on the vaccination history of the included women. The two groups analyzed (women tested 2008–2010 and 2015–2018 respectively) are different in size due to the longer enrollment period of Group 2, and the increased accessibility to the laboratory for the five counties. Even if some data that may influence acceptance of vaccination (religion, education level, socioeconomic status) are still missing, the results facilitate the assessment of seroprevalence and identification of high-risk groups. We cannot completely exclude the fact that some participants were tested in both analyzed periods, but it is expected that the number would be extremely small.

## 5. Conclusions

Overall, rubella virus susceptibility among fertile women from western Romania increased from 2008–2010 to 2015–2018. The highest susceptibility has been in women born between 1997 and 2004 eligible for MMR vaccine through a family practice system and the lowest in the group of women born between 1989 and 1994 eligible for monovalent RCV conducted in schools. No statistically significant difference between the seroprevalence of women in urban and rural areas has been found, either as a whole or in individual cohorts. More efforts should be aimed at the active immunization of fertile women with increased susceptibility to rubella.

## Figures and Tables

**Table 1:** Seroprevalence of rubella among fertile women according to the year of birth and place of residence.

**Group 1 (Tested 2008–2010)**								
	**<1989**		**1989–1994**		**1995–1996**		**>1997**	
Overall % (95% CI)	1336/1422 94.0% (95.1–92.6)	Ref.	30/30 100% (86.6–100)	OR = 3.94 (*p* = 0.25)				
Urban %(95% CI)	977/1038 94.1% (92.5–95.4)	Ref.	8/8 100% (67.6–100)	NA	-		-	
Rural % (95% CI)	359/384 93.5% (90.6–95.6)	Ref.	22/22 100% (85.1–100)	OR = 0.0 (*p* = 0.38)	-		-	
OR (*p* value) ^1^	0.84 (0.52)	-	-	-	-		-	
**Group 2 (Tested 2015–2018)**								
	**<1989**		**1989–1994**		**1995–1996**		**>1997**	
Overall % (95% CI)	2900/3218 90.1% (89.0–91.1)	Ref.	1701/1776 95.8% (94.7–96.6)	OR = 2.48 (*p* < 0.0001)	211/247 85.4% (80.5–89.3)	OR = 0.64 (*p* = 0.02)	182/221 82.4% (76.8–86.8)	OR = 0.51 (*p* = 0.006)
Urban % (95% CI)	2341/2591 90.4% (89.2–91.4)	Ref.	752/776 96.9% (95.4–97.9)	OR = 3.3 (*p* < 0.0001)	87/96 90.6% (83.1–95.0)	OR = 1.03 (*p* > 0.99)	67/80 83.8% (74.2–90.3)	OR = 0.55 (*p* = 0.05)
Rural % (95% CI)	957/1045 91.6% (89.7–93.1)	Ref.	551/582 94.7% (92.5–96.2)	OR = 1.6 (*p* = 0.02)	124/151 82.1% (75.2–87.4)	OR = 0.42 (*p* = 0.0006)	115/141 81.6% (74.4–87.1)	OR = 0.40 (*p* = 0.0006)
OR (*p* value) ^1^	1.16 (0.25)	-	0.56 (0.05)	-	0.47 (0.06)	-	0.85 (0.71)	

^1^ Comparison between urban and rural areas. Ref.= reference; NA = not applicable.

**Table 2:** Rubella seroprevalence among fertile women according to age.

Age (Years)	Group 1 (Tested 2008–2010)		Group 2 (Tested 2015–2018)	
	No. (Positive/Total) % (95% CI)	OR (*p* Value)	No. (Positive/Total) % (95% CI)	OR (*p* Value)
<25	395/417 94.7 (92.1–96.5)	Ref.	1190/1303 91.3 (89.7–92.7)	Ref.
26–34	881/941 93.6 (91.9–95.0)	0.81 (0.46)	2918/3180 91.8 (90.8–92.7)	1.05 (0.63)
>35	90/94 95.7 (89.6–98.3)	1.25 (>0.99)	886/979 90.5 (88.5–92.2)	0.90 (0.50)

## Data Availability

The data sets used and/or analyzed during the present study are available from the last author on reasonable request.
